# Seasonal variation in activity budgets of critically endangered Bornean banded langur, *Presbytischrysomelaschrysomelas* in Malaysian Borneo

**DOI:** 10.3897/BDJ.13.e141783

**Published:** 2025-05-15

**Authors:** Tukiman Nur-Aizatul, Abd Rahman Mohd-Ridwan, Mohammad Noor-Faezah, Roberta Chaya Tawie Tingga, Mohamad Fhaizal Mohamad Bukhori, Jayasilan Mohd-Azlan, Azroie Denel, Muhammad Abu Bakar Abdul-Latiff, Badrul Munir Md-Zain

**Affiliations:** 1 Animal Resource Science and Management, Faculty of Resource Science and Technology, Universiti Malaysia Sarawak, 94300, Kota Samarahan, Malaysia Animal Resource Science and Management, Faculty of Resource Science and Technology, Universiti Malaysia Sarawak, 94300 Kota Samarahan Malaysia; 2 Centre for Pre-University Studies, Universiti Malaysia Sarawak, 94300, Kota Samarahan, Malaysia Centre for Pre-University Studies, Universiti Malaysia Sarawak, 94300 Kota Samarahan Malaysia; 3 Department of Biological Sciences and Biotechnology, Faculty of Science and Technology, Universiti Kebangsaan Malaysia, 43600, Bangi, Malaysia Department of Biological Sciences and Biotechnology, Faculty of Science and Technology, Universiti Kebangsaan Malaysia, 43600 Bangi Malaysia; 4 Institute of Biodiversity and Environmental Conservation, Universiti Malaysia Sarawak, 94300, Kota Samarahan, Malaysia Institute of Biodiversity and Environmental Conservation, Universiti Malaysia Sarawak, 94300 Kota Samarahan Malaysia; 5 Sarawak Forestry Corporation, Kota Sentosa, Kuching, Malaysia Sarawak Forestry Corporation, Kota Sentosa Kuching Malaysia; 6 Environmental Management and Conservation Research Unit (eNCORe), Faculty of Applied Sciences and Technology (FAST), Universiti Tun Hussein Onn Malaysia (Pagoh Campus) 84000, Muar, Malaysia Environmental Management and Conservation Research Unit (eNCORe), Faculty of Applied Sciences and Technology (FAST), Universiti Tun Hussein Onn Malaysia (Pagoh Campus) 84000 Muar Malaysia

**Keywords:** Colobine, behaviour, conservation, Borneo

## Abstract

The activity budget of a primate varies in response to environmental and habitat conditions, including seasonality. To elucidate how primates adapt their behavior to the seasonal shift, the activity budget of Bornean banded langurs (*Presbytischrysomelaschrysomelas*) in the Tanjung Datu National Park southwestern of Sarawak and stratum utilization were studied from July 2023 to February 2024. The behavioral data were collected through scan sampling (336 observation hours) of Bornean banded langur groups. Overall, the langurs allocated most of their time to resting (35.4%), feeding (32.3%), moving (16.2%), vocalizing (9.2%), and social activities like grooming and playing (6.9%). The seasonal variation was evident, with increased foraging and social activities during the dry season and a greater allocation of time to locomotion during the wet season. Additionally, the Mann-Whitney U test revealed significant seasonal fluctuation in social activities (grooming and playing, *P*<0.001), while feeding, moving, and resting remained unaffected (*P*>0.05). Food availability and distribution may influence the langur activity budget, with increased travel distance during the wet season because of food scarcity. The langurs predominantly use stratum C (21–30 m) to feed, rest, move, and socialize. This study highlights the seasonal ecology of this critically endangered endemic primate and underscores the importance of habitat conservation efforts to ensure the sustainability of their populations amid environmental changes.

## Introduction

The activity budget is the allocation of time by animals for different daily activities based on their fundamental ecological requirements, such as feeding, mating, and resting (Borgeaud et al. 2021, Zhou et al. 2022). Understanding primate behavior is essential because it reveals how primates allocate their energy among key activities, including social interactions, resting, and foraging (Dunbar et al. 2009, Decemson et al. 2018). The activity patterns of most primates exhibit time allocation variability. Environmental factors, including weather, food availability and quality, climatic conditions, and anthropogenic factors have significant impacts on their activity budgets (Zhou et al. 2007, Kaplin and William 2013). Furthermore, physiological factors, such as reproductive status, age, energy requirements, body mass, and sex, may influence primate behavior given their critical roles in reproduction and survival (Kaplin and William 2013, Naher et al. 2022).

The spatial and temporal variations in climate affect the habitat structure, resource productivity, and food availability, consequently driving changes in primate activity patterns (Mohamed et al. 2004, Coleman et al. 2021). Research indicates that primate populations adjust their time allocation in response to climatic conditions, with seasonal variations in food availability often leading to significant activity changes (Coleman et al. 2021, Souza‐Alves et al. 2021). During periods of food scarcity, primates may adopt energy-conserving strategies, such as increasing rest, or employ time-maximizing tactics by extending the time and the energy allocated to optimizing foraging opportunities Oates 1987). For example, Phayre’s langurs (*Trachypithecusphayrei*) in semi-evergreen forests increase their travel distance in response to resource shortages (Naher et al. 2022). The seasonal variations in activity budgets have been extensively documented in primates from various regions, including subtropical areas characterized by extreme climatic conditions, such as cold and dry winters (Li et al. 2020, Zhou et al. 2022, Chakravarty and Saikia 2023, Zhang et al. 2023). In other tropical regions, primate behavior variations have been observed between the dry and wet seasons (Pinheiro et al. 2013, Yazezew et al. 2020, Mola et al. 2022).

The Bornean banded langurs (*Presbytischrysomelaschrysomelas*) are elusive and critically endangered primates ([Bibr B12221668][Bibr B12982441][Bibr B12221668], Ampeng et al. 2024). Endemic to Borneo, this species is distinguished by its striking black fur with a white underside on its tail and limbs, a feature particularly notable in the subspecies *P.c.chrysomelas* (Noor-Faezah et al. 2023). Listed as critically endangered and classified as totally protected species in Sarawak under the Wild Life Protection Ordinance of 1998 (WLPO), this primate is particularly sensitive to environmental changes. Historically, this species is common in Sarawak, but their population shrank because of habitat loss from land conversion to oil palm plantations (Nijman et al. 2020). Currently, this arboreal primate is reported to inhabit the lowland, mangrove, and peat swamp forests of Central and Southwestern Sarawak and part of Kalimantan (Phillipps and Phillipps 2018). Given their restricted habitat and sensitivity to environmental changes, comprehending their habitat use is crucial for formulating targeted conservation strategies.

The species started to gain research attention because of their critical conservation status. Up to date, an increasing number of studies covering the occurrence (Ampeng 2003, Rifqi et al. 2019a, Ampeng et al. 2024), sampling technique (Ampeng and Md Zain 2007), ranging behavior (Ampeng and Md-Zain 2012), habitat use (Mustafa and Santoso 2020), diet (Santoso et al. 2023, Tingga et al. 2024), daily activity patterns (Ampeng 2007, Santoso et al. 2023, Nur-Aizatul et al. 2024), and conservation (Rifqi et al. 2019b, Noor-Faezah et al. 2023) of *P.chrysomelas* have been conducted. Information concerning the influence of seasonality toward the activity patterns of primates in Malaysia remains sparse, thus comprehending how *P.chrysomelas* adjust its activity budget between dry and wet seasons is crucial for its conservation and management. Assessing how primates manage their activity budgets on a daily and yearly basis is vital for understanding their environmental interactions and how they effectively use their time and energy to support both reproduction and survival (Defler 1995, Long et al. 2010). This study aims to provide insight into how Bornean banded langurs adjust their activity patterns and adaptation strategies in response to seasonal changes in their natural habitat.

## Material and methods

### Study site

**Figure 1.**
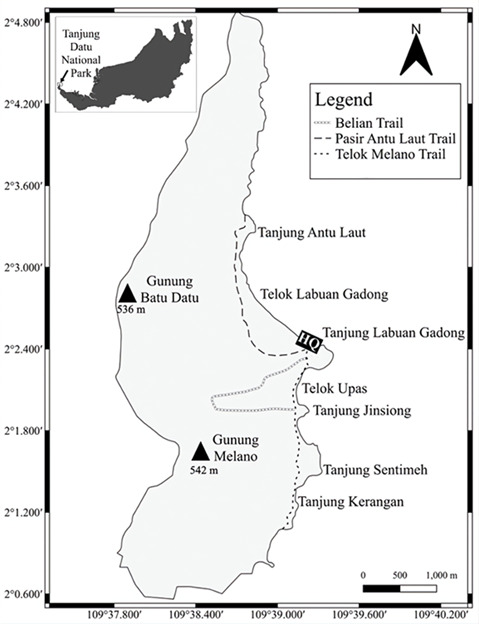
Map of Tanjung Datu National Park

This research was carried out at Tanjung Datu National Park (TDNP) in Lundu District, Sarawak (Fig. 1). The TDNP lies adjacent to the Kalimantan, Indonesia border and has been gazetted in 1994. With an area of approximately 13.79 km², the TDNP is on the edge of the Sarawak southwest region. The area comprises a slender range of hills predominantly covered by a mixed dipterocarp forest with the highest altitude of 543 m a.s.l. (Abdul-Rahman et al. 2015). The annual precipitation in the studied area ranges from 2500 to 4000 mm, indicating a tropical climate. The dry season (southeast monsoon) typically lasts from June to September, while the wet season (northeast monsoon) occurs from November to March, with the highest rainfall recorded in December and January (MET Malaysia 2023).

### Data Collection

The activity budget of *P.c.chrysomelas* was observed using the scan sampling technique for 336 h (168 h for each season) from July 2023 until February 2024. Observations were conducted in the morning (0700–1030) and in the late afternoon (1600–1830). Data collection during the wet season was conducted on non-rainy days to ensure consistent observation conditions and minimize potential observer bias. This approach allowed for reliable comparisons of activity patterns between seasons. Behavioral sampling started when the langurs were first observed and ended when they moved out of observer sight or could no longer be tracked. Any individuals or groups of langurs spotted within 30 m were recorded, including the total number of individuals present. To prevent repeated sampling, the langurs were surveyed in a clockwise or left to right direction at 10 min intervals, with a 5 min rest period between each scan. Langurs were seen at a minimum distance of 5–10 m when feasible. Field protocols emphasized maintaining a consistent distance, avoiding abrupt movements, and limiting noise to reduce observer impact and behavioural disturbance. The langurs were observed more frequently during the dry season (345 min) compared to the wet season (235 min). The mean daily observation time was 15 min of 420 min of sampling effort. Each scan was observed for a minimum of 5 min and a maximum of 70 min.

The frequency of behavior activity of the *P.c.chrysomelas* was recorded during the scan sampling period (Altmann 1974,Bateson and Martin 2021). Behavioral activities were recorded following the categories established by Nur-Aizatul et al. 2024, which included locomotion (walks, running, jumping, and climbing), feeding (eating and chewing food item), resting (being inactive, sleep, sitting, or lying down), vocalizing, grooming, and playing (playful action that can be done alone or with other members involving nonaggressive gesture). While detailed dietary data were not systematically collected, general observations indicated that the langurs primarily consumed foliage, with occasional fruit consumption during the dry season. These observations align with previous studies on colobine primates, which are known to be primarily folivorous but may supplement their diet with fruits when available Matsuda et al. 2009a, Hanya and Bernard 2012). Additionally, the position of each scanned individual was recorded within the following strata to assess the relative use of different strata in the environment: ground level (0–1 m), stratum A (2–10 m), stratum B (11–20 m), stratum C (21–30 m), and stratum D (more than 30 m) (Yap et al. 2019, Santoso et al. 2023).

### Data analysis

The activity budget of the primates was determined by calculating the frequency of each behavioral category using the formula: y = (ny/N) × 100; where, ny represents the amount of records of activity y, and N is the total amount of records obtained during the study period (Pinheiro et al. 2013, Vanaraj and Pragasan 2021). The behavioral frequencies and percentages were documented in Microsoft Excel version 2021. The Shapiro–Wilk test was used to test the data normality. A chi-square test of independence was employed to determine if there were significant differences in the distribution of time allocated to various activities. Non-parametric Mann–Whitney U test was used to compare the seasonal variation activity patterns of langurs in the wet and dry seasons. All statistical analyses were performed using PAST software (Hammer et al. 2001).

## Results

### Diurnal activity budget

**Figure 2.**
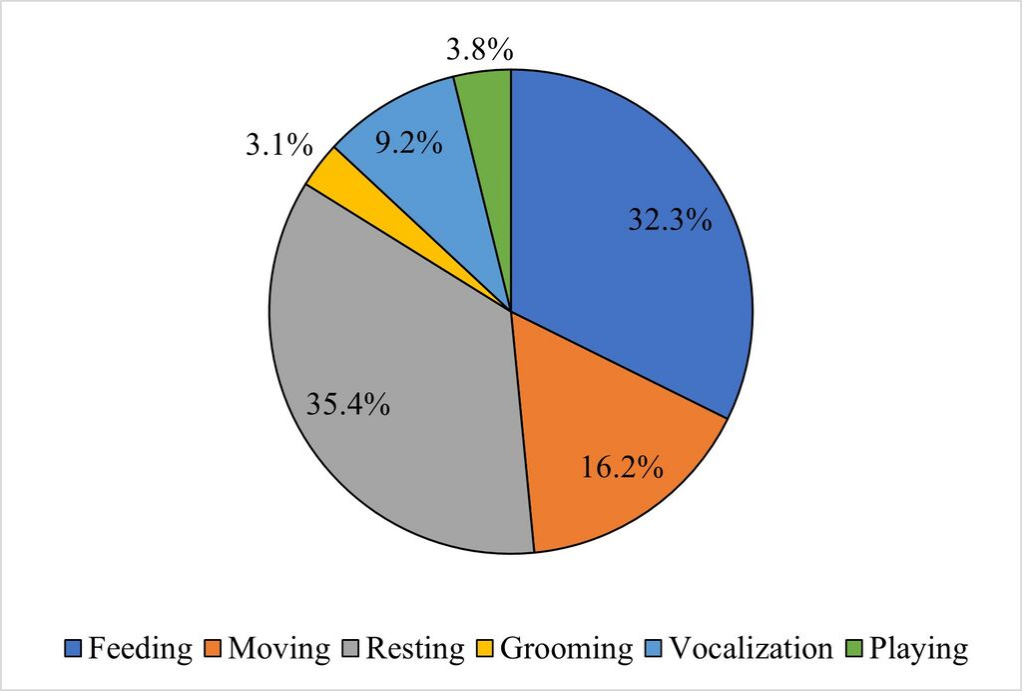
Percentages of daily activity budget of Bornean banded langur recorded in TDNP, Sarawak.

Analysis of 130 observational scans revealed that *P.c.chrysomelas* allocated the majority of their daily activity budget to resting (35.4%) and feeding (32.3%), with social behaviors such as grooming and playing comprising a notably smaller proportion (6.9%) (Fig. 2). A chi-square test of independence confirmed significant differences in the distribution of these activities (x^2^=77.95, df=5, *P* < 0.001).

### Seasonal activity budget

Activity budgets of *P.c.chrysomelas* showed distinct seasonal trends (Table 1). Although statistical analysis indicated no significant seasonal differences in feeding (U = 3, z = 0.231, *P* = 0.825), resting (U = 3, z = 0.436, *P* = 0.663), moving (U = 3, z = 0.449, *P* = 0.653), and vocalizing (U = 4, z = 0, *P* = 1.0), notable numerical variations were recorded. Feeding activity occurred more frequently during the dry season (38.9%), as opposed to the wet season (24.1%), as was resting (38.9% in the dry season versus 31.0% in the wet season). In contrast, moving and vocalizing activities were more prevalent during the wet season (29.3% and 12.1%, respectively) compared to the dry season (5.5% and 6.9%, respectively). Social activities demonstrated a statistically significant seasonal fluctuation (*P* < 0.001), with grooming and playing occurring more frequently during the dry season than the wet season (Table 1).

### Strata use

**Figure 3.**
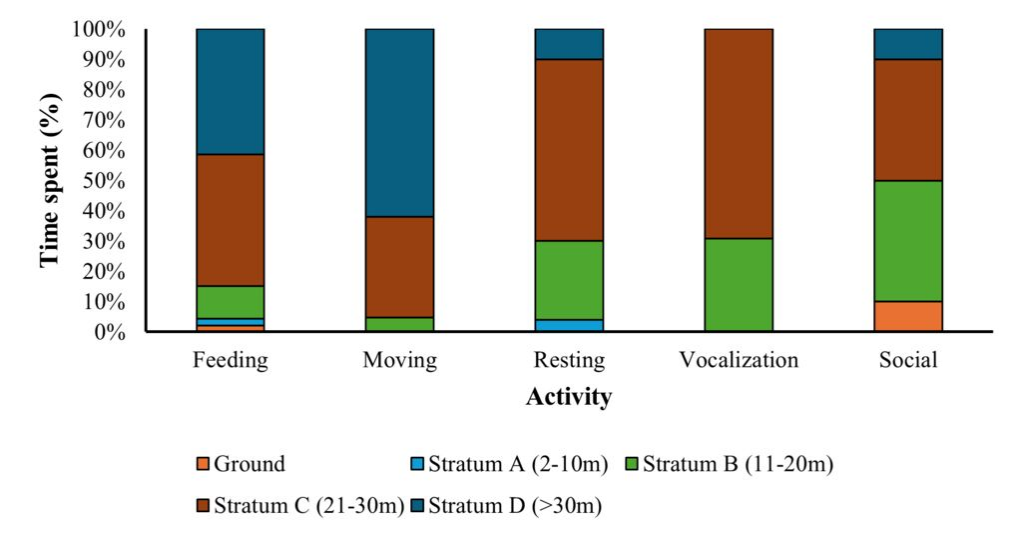
The percentage of time spent in different activities across various strata by Bornean banded langur.

**Figure 4.**
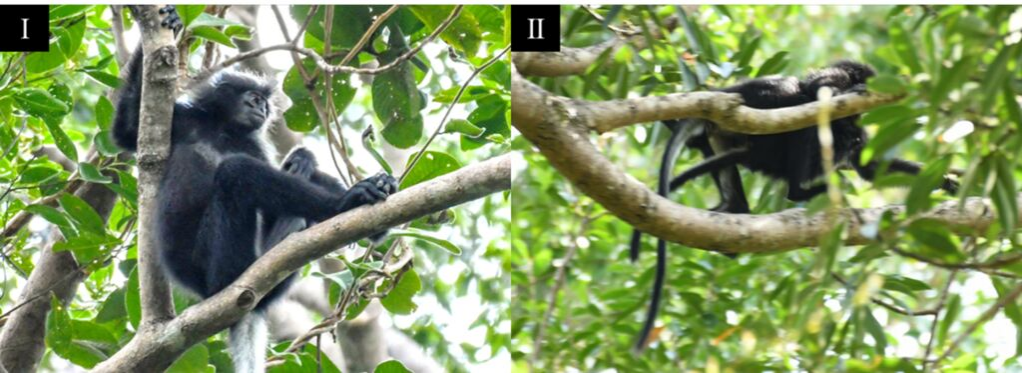
(I) Adult male Bornean banded langur resting on the tree branch. (II) Adult female Bornean banded langur carrying its infant.

The TDNP was dominated by mixed dipterocarp forests with tall trees that are more than 20 m with a verdant canopy. The results showed that Bornean banded langurs primarily occupied heights between 21 and 30 m (stratum C) when engaging with most activities (Fig. 3). However, they utilized the higher canopy level (stratum D) (61.9%) when moving, particularly when perceiving a threat. Langurs spent half of their time doing social activities, most particularly playing, grooming, and other activities at strata B and C. The langurs also spent minimal time in feeding (10.9%) and resting (26%) on small trees (<10 m) (stratum A) (Fig. 4). Occasionally, the langurs were found feeding and socializing (i.e., playing) on the ground (2.2% and 10%, respectively).

## Discussion

In this study, *P.c.chrysomelas* exhibited the most prevalent behavior of resting, followed by feeding and moving, which were consistent with observations reported for other Asian primates, such as *Nasalislarvatus* (Matsuda et al. 2009b, Iskandara et al. 2016), *P.rubicunda* (Maklarin 2008), *T.auratus* (Fitriyani and Purba 2023) and *T.francoisi* (Le et al. 2024). The Bornean banded langurs also showed variations in their seasonal activity patterns, typically starting the day between 0600 and 0630 and ending it around 1900. However, during the wet season, langurs daily activities shifted to a later start and an earlier return to the sleeping site often at around 1730.

The Bornean banded langurs exhibited seasonal variation in their activity budgets, with significant differences observed in social activities such as grooming and playing (*P* < 0.001), with higher frequencies observed during the dry season. This finding is supported by a similar observation in *Plecturocebuscaquentensis* (Carolina et al. 2024) and *Colobusguereza* (Shumet and Yihune 2017), which also exhibit increased grooming activities during the dry season. However, this finding is in contrast with that of other studies reporting higher levels of social engagement during the wet season (Long et al. 2010, Yazezew et al. 2020). Although grooming is typically more common during the wet season to conserve energy, this behavior is not observed in *P.c.chrysomelas* during the wet season. The reduction in grooming activities may be caused by intense food competition within the group, as reported in free-ranging *M.mulatta* (Loy 1970, Wubs et al. 2018) and baboons (Alberts et al. 2005).

While feeding (U = 3, z = 0.231, *P* = 0.825) and resting (U = 3, z = 0.436, *P* = 0.663) did not show significant seasonal variation, descriptive data indicate a trend toward increased feeding during the dry season (38.9%) compared to the wet season (24.1%). The increased feeding activity during the dry season is likely associated with the abundance and quality of food resources. As reported for other colobines in Sabah, the feeding time of *P.rubicunda* and *N.larvatus* coincides with periods of fruit abundance (Matsuda et al. 2009a, Hanya and Bernard 2012, Matsuda et al. 2014). However, *P.c.chrysomelas* are primarily folivores; hence, the increased feeding activity during dry season may reflect a strategy to compensate for lower quality foliage, similar to behaviors observed in *T.francoisi* (Zhou et al. 2007) and *T.poliocephalus* (Hendershott et al. 2016), which also consume lower-quality fallback foods during the dry season. While the current study did not systematically collect data of detailed diets, other previous studies of colobines suggest that fruits constitute a small but important part of their diet, notably in periods of abundance (Santoso et al. 2023; Nur-Aizatul et al. 2024).

Although the Mann-Whitney U test did not reveal significant differences in locomotion between seasons (U = 3, z = 0.449, *P* = 0.653), the descriptive data suggest a notable increase in movement during the wet season (29.3%) compared to the dry season (5.5%). Similar to that reported for *Callithrixflaviceps* (Souza‐Alves et al. 2011) and *P.caquetensis* in Colombia (Carolina et al. 2024), suggesting that the high locomotion during the rainy season is caused by the scarcity and the scattered distribution of resources. This situation forces the primate to travel further to meet nutritional needs and expand their home range in the lean season (Yazezew et al. 2020). Deng et al. (2023) similarly observed a significant increase in the home range and daily travel distance of *T.francoisi* due to food scarcity. In contrast, the travel distance of *P.c.chrysomelas* in the Samunsam Wildlife Sanctuary was not affected by the availability of resources because the langurs did not experience food scarcity (Ampeng and Md-Zain 2012). However, *P.c.chrysomelas* occasionally travel further when preferred food (e.g., fruits) was more dispersed (Ampeng and Md-Zain 2012). In Sabah, *N.larvatus* also exhibited shorter daily travel distances during the fruit abundance season as the species prefers the fruits of dominant tree plant species (Matsuda et al. 2009a). Additionally, tropical primates are typically less active and spend less time in moving during the dry season as a strategy of reducing energy expenditure (Asensio et al. 2009, Masi et al. 2009, Dunbar et al. 2019).

In this study, *P.c.chrysomelas* exhibited behavioral flexibility by adopting both energy-conservation and energy maximizing strategies in response to seasonal changes in food availability. During the dry season, when fruits and other high-quality foods were more abundant, langurs increased their feeding activity (38.9%) and engaged in more social behaviors such as grooming (5.6%) and playing (4.2%). This energy-maximizing strategy allows them to exploit resource abundance, similar to patterns observed in *Semnopithcuspriam* in India (Mola et al. 2022) and *Colobusangolensisruwenzorii* in Uganda (Arseneau-Robar et al. 2020), which are known as energy maximizers. These behaviors not only enhance energy intake but also facilitate social bonding in the group, which are crucial for group cohesion and individual fitness (Dunbar 2010, Jablonski 2021).

Conversely, during the wet season, when resources are scarce, *P.c.chrysomelas* spent less time in feeding (24.1%) and rested more (31.0%), suggesting a shift toward energy-conservation strategies. Due to its complex foregut digestive system, langurs undergo a slow fermentation process, requiring extended resting periods and reduced energy expenditure when food is scarce (Oates 1987, Dasilva 1992, Dunbar 1992, Arseneau-Robar et al. 2020). Similar energy-conservation strategies have been reported in *P.femoralis* during non-fruiting periods (Najmuddin et al. 2020). By minimizing energy expenditure and increasing resting periods, langurs can optimize nutrient absorption and survive periods of food scarcity. The ability of *P.c.chrysomelas* to shifts between these strategies highlights their behavioral adaptability in response to seasonal fluctuations in food availability.

Moreover, the influence of the seasonal variation on the social activities of *P.c.chrysomelas* is not yet fully understood. Therefore, further research should investigate the role of food competition and resource availability in shaping these seasonal behaviors of *P.c.chrysomelas*. While efforts were made to standardize observation conditions, the limited sampling window during the wet season may have introduced some observer bias. Future studies should aim to collect data across a broader range of weather conditions to better understand the influence of rainfall on activity patterns. Additionally, the small sample size in this study may have limited the statistical power to detect significant differences in some activities, such as locomotion, despite the observed increase during the wet season. Thus, future studies with larger sample sizes and detailed dietary data are needed to confirm these patterns and better understand the ecological drivers of seasonal behavior in this critically endangered species. Understanding these dynamics will be crucial for developing effective conservation strategies.

## Conclusions

This study provides valuable insights into the seasonal activity patterns of *P.c.chrysomelas*. Our findings reveals that the Bornean banded langur activity patterns in TDNP vary across dry and wet seasons. Major activities such as feeding, resting, and moving showed no significant differences between seasons. However, social activities, including grooming, playing, and other interactions, varied significantly across the seasons. Our findings confirm that the Bornean banded langur can adjust its social activity patterns to cope with environmental shift in tropical rainforests and during wet season. This underscores the importance of understanding seasonal influences on primate behavior to develop effective conservation strategies. Additionally, this study emphasized the critical role of the forest canopy in providing langurs with protection, dietary resources, and shelter. As the langurs face the threats posed by habitat loss, a well-preserved canopy is critical to sustaining the microhabitats required for their survival. Furthermore, these findings enhance the understanding of primate behavioral adaptations to seasonal fluctuations in their habitat, highlighting the intricate relationship between ecological conditions and activity patterns. Further research is required to investigate how food competition, resource availability, and social dynamics interact to influence these seasonal behaviors.

## Figures and Tables

**Table 1.** Number of activity frequency and mean activity time budget (%) of Bornean banded langur during the dry and wet seasons in TDNP, Sarawak. Notes: '*’ – statistically significant.

**Activity**	**Dry**		**Wet**		**The overall no. of activity frequency**	**Total activity budget (%) ± *SE***	***P* value**
	No. of activity frequency	Activity budget (%) ± *SE*	No. of activity frequency	Activity budget (%) ± *SE*			
**Feeding**	28	38.9% ± 0.13	14	24.1% ± 0.09	42	32.3% ± 0.104	0.825
**Moving**	4	5.5% ± 0.02	17	29.3% ± 0.13	21	16.2% ± 0.17	0.653
**Resting**	28	38.9% ± 0.16	18	31.0% ± 0.14	46	35.4% ± 0.05	0.663
Grooming*	4	5.6% ± 0.02	0	0.0%	4	3.1% ± 0.04	*P*<0.001
**Vocalizing**	5	6.9% ± 0.03	7	12.1% ± 0.07	12	9.2% ± 0.04	1.0
Playing*	3	4.2% ± 0.02	2	3.5% ± 0.02	5	3.8% ± 0.005	*P*<0.001
**Total**	**72**	**100**%	**58**	**100**%	**130**	**100**%	
